# Establishment of a gastric cancer subline with high metastatic potential using a novel microfluidic system

**DOI:** 10.1038/srep38376

**Published:** 2016-12-05

**Authors:** Zhe-zhou Chen, Wan-ming Li, Yu Zhang, Min Yu, Lian-feng Shan, De-zheng Yuan, Fu-rong Liu, Jin Fang

**Affiliations:** 1 Department of Cell Biology, Key Laboratory of Cell Biology, Ministry of Public Health, and Key Laboratory of Medical Cell Biology, Ministry of Education, China Medical University, Shenyang 110122, PR China; 2 Department of Mathematics, China Medical University, Shenyang 110122, PR China

## Abstract

Metastasis is an important hallmark of malignant tumors. In this study, we developed a microfluidic system to screen highly metastatic sublines via differential resolution of cell invasiveness. The system was composed of a PDMS-glass device connected with a syringe pump and a Petri dish. To facilitate the selection process, a long-term cell invasion driving force based on a chemotactic factor gradient was created using the Petri dish-based liquid supply pattern, and the invasive cells were collected for round-by-round selection via an open region in the chip. Using the system, we established an SGC-7901/B2 subline from the human gastric cancer SGC-7901 cell line by only two rounds of selection. *In vitro* assays showed that the SGC-7901/B2 cells were superior to the parental cells in proliferation and invasiveness. Furthermore, an *in vivo* tumorigenicity assay demonstrated that compared with the parental cells, the subline had stronger spontaneous metastatic and proliferative capability, which led to a shorter survival duration. Additionally, the protein expression differences including E-cadherin and Smad3 between the subline and parental cells were revealed. In conclusion, this microfluidic system is a highly effective tool for selecting highly metastatic sublines, and SGC-7901/B2 cells could serve as a potential model for tumor metastasis research.

Metastasis is an important hallmark of malignant tumors, and it is responsible for more than 90% of cancer-related patient death^1^. For gastric cancer, nearly 50% of newly diagnosed patients suffer from metastases, and this leads to poor prognoses and high mortality rates^2^.

Numerous reports have demonstrated that tumors are highly heterogeneous, and only a small number of subpopulations within a primary tumor have the potential to invade across the basal membrane and finally metastasize to distant organs^3^,^4^,^5^,^6^. Therefore, selection and characterization of such highly metastatic subpopulations are crucial for understanding metastatic mechanisms, discovering new therapeutic targets, and screening metastasis-suppressing anticancer drugs. Currently, the *in vivo* orthotopic implantation model is the most widely used method to establish highly metastatic sublines^7^,^8^,^9^,^10^. Tumor cells are transplanted into nude mice and the metastatic subpopulation is taken out and cultured. After several cycles, the cell sublines with higher metastatic potentials are established. Although this *in vivo* selection technique has the advantage of organ-specificity, it is limited by operation complexity, time and cost consumption. It can also be easily influenced by hosts due to individual differences. Consequently, *in vitro* selection technologies, including transwell assays, have emerged^11^,^12^,^13^. Using transwell chambers, the subline selection can be carried out based on the different abilities of cells to migrate through a Matrigel-coated polycarbonate membrane by a chemotactic factor gradient driving force. Compared with *in vivo* methods, this is relatively simple and not influenced by the hosts. However, the driving force to facilitate cell migration cannot be continuously maintained during the selection owing to time-dependent fading of the chemotactic factor gradient, resulting in a limited capability to resolve and select differentially invasive cells^14^. Thus, an effective, simple approach with high resolution and specificity is highly desirable.

Recently, microfluidic platforms have been broadly exploited in biomedical fields due to their advantages, including small sample consumption, high automation and integration, and realistic *in vivo* microenvironment recapitulation; a particular advantage is ease of liquid handing, which enables long-term cell culture for cell-related analyses. Many microfluidic devices have been developed to investigate tumor invasion and metastasis based on cleverly designed microchannels that mimic the metastasis microenvironment^15^,^16^,^17^,^18^,^19^,^20^,^21^,^22^. However, to our knowledge, none of them have been used to screen highly metastatic sublines, in part because of their inability to collect selected cells and maintain cell migration-driving forces long-term for resolving cell invasion differentiation.

Accordingly, we developed a novel microfluidic system to screen highly metastatic sublines by designing an open region for selected cell collection and a Petri dish-based liquid supply system to establish a long-term cell migration driving force. With this platform, a highly metastatic subline derived from the human gastric cancer SGC-7901 cell line was established by only two selection cycles. The *in vitro* and *in vivo* assays all demonstrated that this subline had more malignant and metastatic potential.

## Results

### Function characterization of the microfluidic system

**Figure 1:**
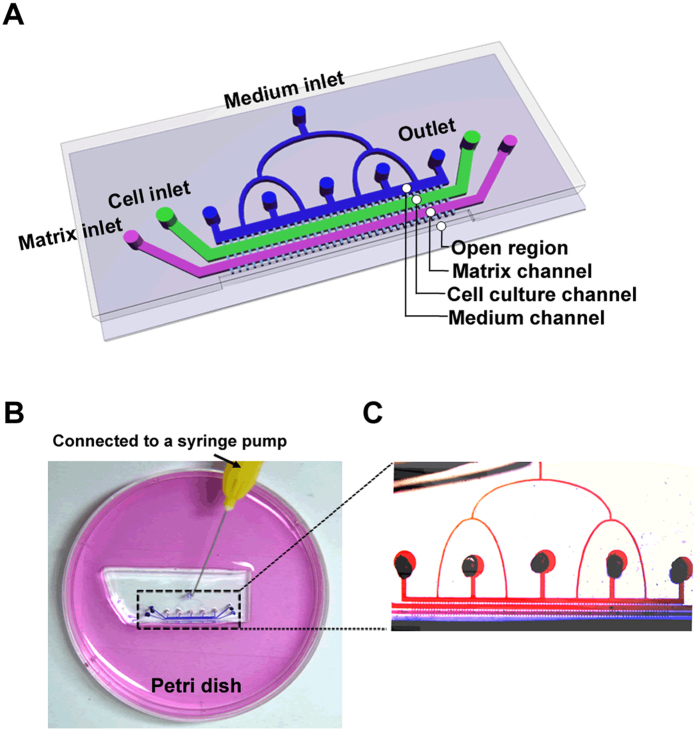
The microfluidic system for subline selection. (**A**) A schematic illustration of the microfluidic device. (**B**) A photograph of the microfluidic system comprising a PDMS-glass chip and a Petri dish with culture medium. (**C**) An image showing a continuous chemical gradient by red and blue dye inside the device using the designed liquid supply system.

The PDMS-glass microfluidic device (Fig. 1A) mainly contained four microchannels—a medium channel, a cell culture channel, a matrix channel and an open region. The adjacent channels were connected by narrow gaps. A low concentration of FBS (2%) was perfused through the medium inlet. A high concentration of FBS (10%) was supplied by the open region. Thus, a concentration gradient could be formed. More details could be seen in the method section.

**Figure 2:**
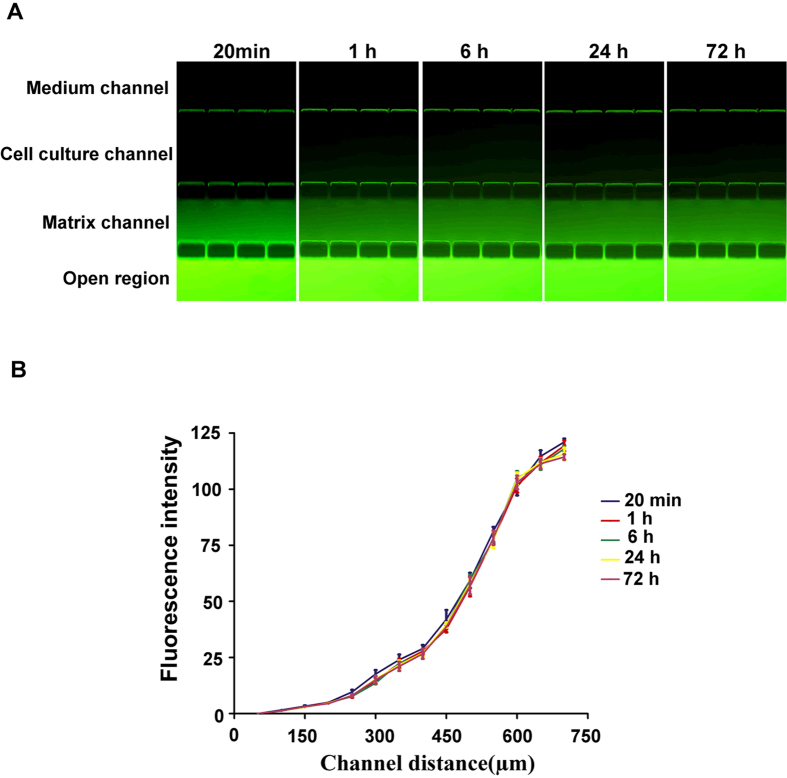
Analysis of concentration gradient generation and maintenance in the channels. The fluorescent dye FITC was used to form a concentration gradient across the channels by pumping PBS without FITC into the medium channel at 3 μL/h and supplying the PBS containing FITC into the open region by a Petri dish. (**A**) Fluorescence gradient images after perfusion of FITC for 20 min, 1 h, 6 h, 24 h and 72 h. (**B**) A quantitative profile of fluorescence intensity across the channels at these time points (original magnification ×200).

To investigate whether the microfluidic system was able to create the concentration gradient required for subline selection, the fluorescent dye FITC was used to evaluate gradient formation and maintenance. As shown in Fig. 2A, under a flow rate of 3 μL/h, a visible fluorescence intensity gradient from the medium channel to the open region was formed in only 20 minutes and was maintained for 72 h. The quantitative analysis showed a continuous intensity gradient along the channels at different time points (Fig. 2B). This result indicated that our system was able to obtain a stable concentration gradient very soon after initiation and that the gradient could be maintained for a long time with a single pump.

**Figure 3:**
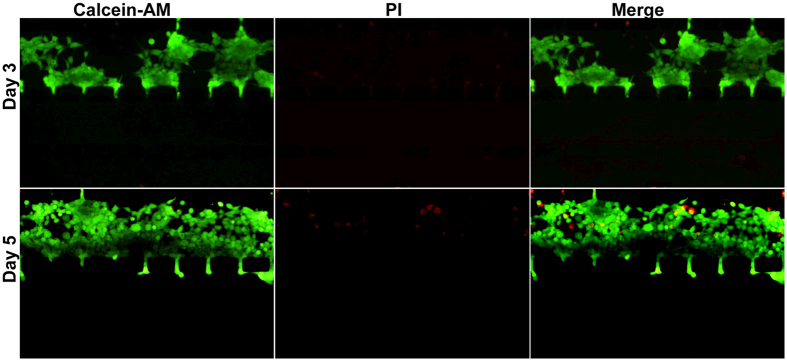
Cell viability assay within the device. Human embryonic kidney HEK-293 cells were seeded and grown in the cell culture channel of the Matrigel-loaded device under the 2–10% FBS gradient with a flow rate of 3 μL/h. After 3 days (upper) and 5 days (lower) of culture, the cells were subjected to live/dead staining. Living cells were stained green by calcein-AM, and dead cells were stained red by PI (original magnification ×100).

Furthermore, to evaluate whether cells could grow normally under the conditions above, we performed a cell viability assay using the HEK-293 human embryonic kidney cell line and a live/dead staining kit. As shown in Fig. 3, after having been cultured in the channel for 3 days, nearly all cells were stained green, indicating they were alive. After continuous culture for 5 days, despite the few cells that were dead (stained red), most of the cells still fluoresced green, and the living cell number had obviously increased compared with the count after 3 days of culture. These results indicated that cells could maintain survival and proliferation in our system for a long time.

### Screening of highly metastatic subline

**Figure 4:**
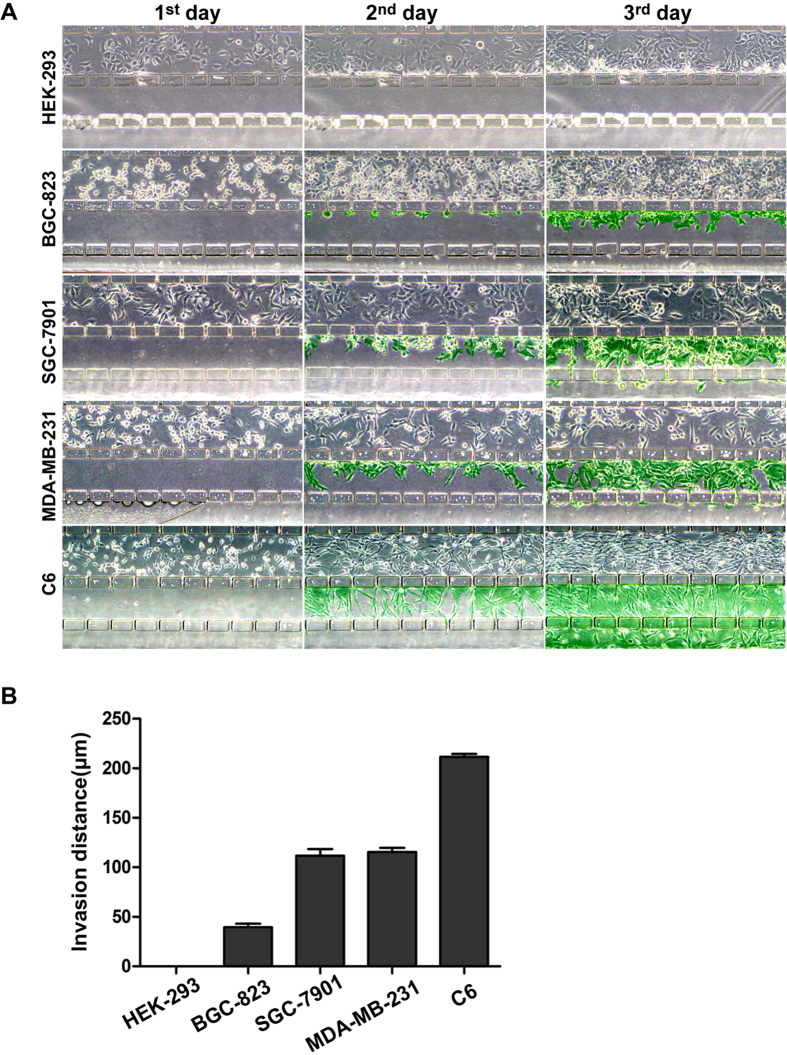
Evaluation of the ability of the microfluidic system to resolve cell invasiveness. Five different types of cells (C6, MDA-MB-231, SGC-7901, BGC-823 and HEK-293) were cultured in the Matrigel-loaded device under a 2~10% FBS gradient with a flow rate of 3 μL/h. **(A)** Images of cell invasion were taken every day by phase-contrast microscopy. The cells invading out of the culture channel are indicated by green. **(B)** The invasiveness of different cell lines was evaluated quantitatively according to the invasion distance of the leading cells (original magnification ×100). Each independent experiment was repeated three times.

Considering our aim to select sublines based on differences in cell invasiveness, we evaluated whether the system had the ability to distinguish the different invasive potentials of various cell lines. As shown in Fig. 4A, HEK-293 cells, derived from normal embryonic kidney cells, did not invade the matrix channel during the 3-day culture period, indicating a lack of invasiveness. In contrast, human gastric cancer BGC-823 cells began to invade through the gaps on day 1, and they migrated into the matrix channel on day 2, revealing a limited invasiveness. However, the other three cell lines, derived from highly metastatic tumor tissues, SGC-7901, MDA-MB-231 and C6, displayed much more invasive cell numbers and migration distances in the matrix channels on the second day and especially on the third day. The invasive ability of these cells was quantified based on the invasion distance of the leading cells in the channels, resulting in an order of C6 > MDA-MB-231 > SGC-7901 > BGC-823 > HEK-293 (Fig. 4B), which is in agreement with the reported invasive properties of these cells^23^,^24^,^25^,^26^,^27^.

In order to further verify the reliability of our approach in invasiveness evaluation, different numbers of SGC-7901 cells were injected into the cell culture channel, respectively. Although difference in the initial cell number, the distances of leading cells were nearly the same (Supplementary Figure S1) which indicated that our system could distinguish invasiveness based on the leading cell invasion. To investigate the possible effect of cell proliferation on invasion ability, we chose two cell lines (BGC-823 and MDA-MB-231) to perform MTS assay. Proliferation of BGC-823 was stronger than that of MDA-MB-231 (Supplementary Figure S2), which was the same as shown in Fig. 4A, with more cell numbers in the cell channel, while the invasion was much weaker than that of MDA-MB-231 evaluated by our system (Fig. 4B). These results indicated that our designed microfluidic system and the evaluation approach based on the leading cell invasion had an excellent resolution of invasiveness without the interference of cell proliferation or cell density.

**Figure 5:**
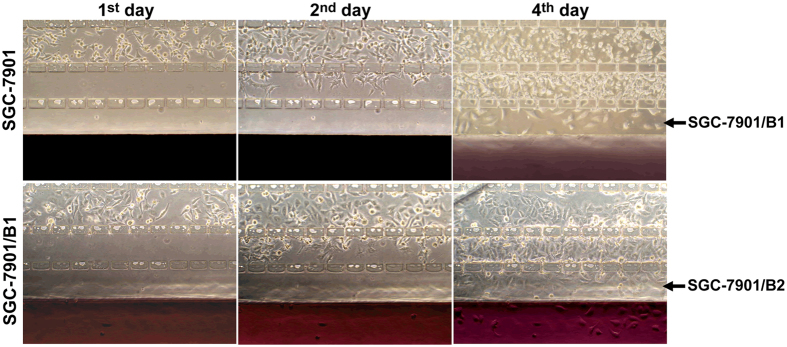
Selection process of the subline from the SGC-7901 cell line. Upper: the first round of selection. Parental cells were seeded into the cell channel. After 4 days, the cells that had invaded the open region (indicated by the arrow) were harvested and named SGC-7901/B1. Lower: the second round of selection. SGC-7901/B1 cells were re-seeded into the cell culture channel and subjected to the same selection. After 4 days of culture and invasion, the achieved subline cells used for amplification culture were named SGC-7901/B2 (original magnification ×100).

Based on the excellent ability of the microfluidic system to resolve the invasiveness of different types of cells, we employed it to screen highly metastatic subline cells from human gastric cancer SGC-7901 cells. The parental SGC-7901 cells were seeded in the culture channel and driven to migrate along the chemotactic factor gradient (from 2% FBS to 10% FBS). As shown in Fig. 5 (upper), during four days of continuous culture, nearly 8% of the seeded cells invaded the matrix channel first and the open region next. These invasive cells were harvested and named SGC-7901/B1 after amplification culturing for about ten days. The SGC-7901/B1 cells were re-seeded and subjected to a second selection round to achieve the SGC-7901/B2 subline. In this cycle, nearly 20% of seeded cells migrated into the open region and were harvested (Fig. 5, lower), which indicated that a highly invasive subpopulation had been enriched through this system.

### Characterization of highly metastatic subline *in vitro*

**Figure 6:**
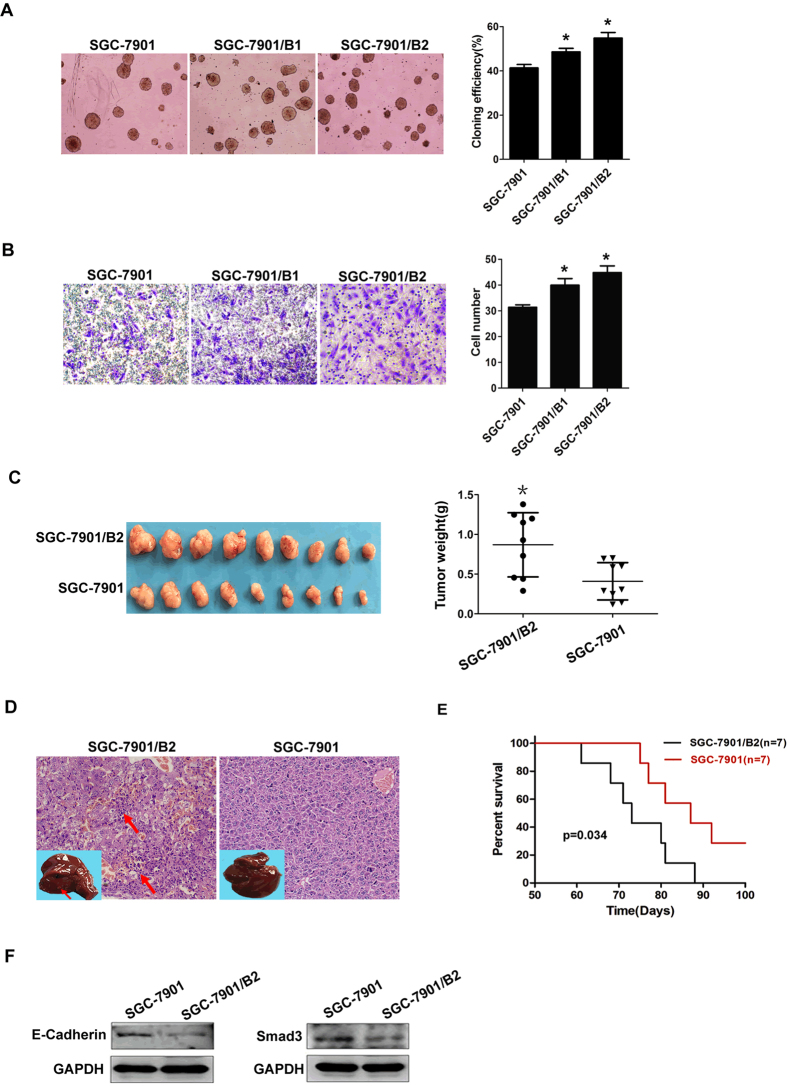
*In vitro* and *in vivo* characteristics of the subline cells. (**A**) Colony formation assay. Parental SGC-7901, SGC-7901/B1 and subline SGC-7901/B2 cells were suspended in soft agar and cultured for two weeks. Colonies were imaged by phase-contrast microscopy (left), and those containing more than 50 cells were quantitatively assessed (right). Each independent experiment was repeated three times. (**B**) Transwell invasion assay. Parental SGC-7901, SGC-7901/B1 and subline SGC-7901/B2 cells were seeded on the upper chamber. The invading cells were stained by Trypan Blue (left) and counted for quantitative analysis (right). Each independent experiment was repeated three times. (**C**) Parental or subline cells (2 × 10^6^) were subcutaneously injected into nude mice. The locally formed tumors were measured by size and weight. (**D**) Representative image of H&E-stained liver sections of mice, and the metastatic nests of gastric cancer subline cells are indicated by arrows. The insets showed liver tissues with metastatic foci indicated by arrows. (**E**) A Kaplan-Meier plot comparing survival durations in mice injected with parental cells and subline cells (1 × 10^6^) (original magnification ×200, *p ≤ 0.05 compared with parental cells). (**F**) Protein expression analysis. Total proteins were extracted from parental and subline cells, respectively and subjected to Western blotting for E-Cadherin and Smad3 protein expression analysis. GAPDH was used as the control protein.

Colony formation assays and transwell invasion analysis were used to evaluate the *in vitro* proliferation and invasion abilities of the sublines, respectively. The colony formation assays (Fig. 6A) showed that the colony formation percentages of the subline SGC-7901/B2 cells (54.8 ± 2.5%) and SGC-7901/B1(48.5 ± 1.6%) were both significantly higher than that of the parental cells (41.3 ± 1.5%) and SGC-7901/B2 were slightly higher than that of SGC-7901/B1 cells, implying a stronger *in vitro* growth ability of the sublines. Transwell analysis (Fig. 6B) demonstrated that the numbers of subline SGC-7901/B2 (44.8 ± 2.6) and SGC-7901/B1(39.9 ± 2.6) cells invading through the Matrigel were both significantly higher than that of parental cells (31.3 ± 1.0), and also, the invading subline SGC-7901/B2 cells were slightly more than that of SGC-7901/B1, which indicated that the subline cells had a stronger *in vitro* invasion ability than the parental cells. Moreover, the subline SGC-7901/B2 cells maintained their invasive potential after ten passages as detected by transwell assay (Supplementary Figure S3).

### Characterization of highly metastatic subline *in vivo*

Considering that the *in vitro* invasiveness of SGC-7901/B2 was superior to that of SGC-7901/B1, we performed the *in vivo* evaluation by using the subline SGC-7901/B2 and its parental SGC-7901 cells. SGC-7901 and SGC-7901/B2 cells were injected subcutaneously into nude mice. The subcutaneous tumors and the potential metastatic organs were measured. The subcutaneous tumors formed by the subline cells were significantly larger in size and weight than those of the parental cells (Fig. 6C). The above data indicated that the subline cells had an enhanced *in vivo* proliferation potential.

A series of organs were excised for evaluation. Metastases in lungs, kidneys and lymph nodes were not found by macroscopic or microscopic examination, but visible liver metastases were observed (Fig. 6D). The metastatic rate resulting from the subline cells (7/9, 77.8%) was significantly higher than that from the parental cells (2/9, 22.2%). The metastatic foci stained by H&E revealed several diffused nests of gastric cancer cells with polymorphic nuclei (Fig. 6D), which further corroborated the gross observations. This indicated that the subline had a stronger spontaneous metastatic ability with a higher metastatic rate to the liver. Survival analysis was performed by tail-vein injection, and a Kaplan-Meier plot showed that mice injected with the subline cells had a noticeably shorter survival period than those injected with parental cells (Fig. 6E), which indicated that the subline cells had a more malignant character.

### Molecular change evaluation

Based on the results of the *in vitro* and *in vivo* assays, molecular changes, including mRNA and protein changes, were further evaluated. The mRNA were extracted from both the parental and subline cells and subjected to gene expression profiling (data not shown). Among the 28,218 analyzed genes, 2,015 genes were up-regulated and 1,729 genes were down-regulated in the subline cells compared with the parental cells, including adhesion-, metastasis-, proliferation- and metabolism-related genes, such as E-cadherin.

The protein expression of E-cadherin and Smad3 was further analyzed by Western blotting (Fig. 6F). Down-regulation of E-cadherin and Smad3 proteins in the subline cells was observed, which was consistent with the results of the gene expression profile. These results all suggested that SGC-7901/B2 had a more malignant and metastatic potential at the molecular level.

## Discussion

Here, we isolated a highly metastatic subline from the biologically heterogeneous human gastric cancer SGC-7901 cell line using our newly designed microfluidic system. The *in vitro* colony formation assays suggested that the SGC-7901/B2 subline cells had an enhanced proliferation potential, which was further confirmed with *in vivo* tumorigenesis analysis by the generation of a greater number of larger tumor masses compared with those generated by the parental cells. More importantly, both the *in vitro* and *in vivo* assays demonstrated that the subline cells possessed a stronger invasion potential compared with that of the parental cells. Transwell analysis showed that the number of subline cells that had invaded through the Matrigel was significantly greater than that of the parental cells. The *in vivo* metastasis assay revealed that the subline cells could evoke a significantly higher liver metastases rate than the parental cells could, which led to an exceedingly high mortality rate.

Apart from the differences between the parental and subline cells in terms of biological phenotype, their molecular differences were further evaluated. Transcriptional profiling analysis revealed that there were 3,744 highly differentially expressed genes (fold change ≥ 2.5), including metastasis-, proliferation-, adhesion-, apoptosis- and metabolism-related genes. Among them, E-cadherin and Smad3 were further detected in protein expression levels by Western blotting. E-cadherin is a crucial cell adhesion molecule and is considered to be a decisive inhibitor for tumor metastasis^1^,^2^,^28^,^29^. Smad3 is a key molecule of the TGF-β/Smad signaling pathway. A series of studies has demonstrated that down-regulated Smad3 could interrupt the normal TGF-β pathway and lead to the loss of the tumor-suppression function^30^,^31^. Both E-cadherin and Smad3 were down-regulated in the subline cells at the transcriptional and translational level, which indicated that the subline had a stronger metastatic potential than the parental cells. Taken together, the above analyses all suggested that SGC-7901/B2 is a subline with high metastatic potential.

Compared with other *in vivo* and *in vitro* methods for subline selection, our system has an excellent selection efficiency. Bai *et al*.^10^ obtained a highly metastatic gastric cancer cell line by four rounds of *in vivo* stepwise selection using an orthotopic tumor cell implantation method. Tie *et al*.^11^ selected highly invasive gastric cancer sublines using 10 rounds of repetitive transwell assays *in vitro*. In our study, only two rounds of selection were required to obtain the metastatic subline. It has been reported that the SGC-7901 cell line itself possesses a highly metastatic phenotype^26^; thus rapidly achieving subline from it with an excessive metastasis potential revealed that this method had a very high selection ability based on cell invasiveness differentiation. In addition, we selected a highly metastatic subline from a lung cancer cell line by using this system (data not shown), and the differences of *in vitro, in vivo* and molecular characteristics including microRNAs and proteins between the subline and the parental cells were demonstrated, which indicated that our system could be widely used in metastatic subline screening. We attribute this selection ability to the capability of the system to stabilize the chemotactic factor gradient for a long time, which leads to a continuous driving for cell migration. The widely used transwell chamber generates a concentration gradient via the passive diffusion of solute from the upper layer to the lower layer, which suffers from a time-dependent loss due to the absence of continuous liquid replacement, resulting in limited cell migration driving forces. In contrast, in our system, a long-term chemotactic factor gradient can be created by adding high concentration FBS in a Petri dish and continuously pumping a low concentration of FBS into the medium channel. Because the volume of medium in the dish was much larger than that of the pumping medium, a stable FBS concentration gradient from the medium channel to the open region could be maintained for cell migration over a long period of time using a single syringe pump. The fluorescent intensity of different microchannels was constant over the course of 72 h, indicating the presence of a driving force during the subline selection.

Here, we designed a continuous liquid supply system to maintain the concentration gradient; however, several studies have demonstrated that flow shear force might influence cell survival or proliferation^32^,^33^. To avoid this interference, we designed multiple outlets in the medium channel that enabled the medium to enter the cell culture channel by liquid diffusion and not by direct flow perfusion, reducing possible shear force. Cell live/dead and invasion assays showed that the cells could maintain normal proliferation and migration phenotypes during the selection period.

Although many microfluidic systems have been reported in tumor invasion research, no attention has been directed toward subline selection, partly because of a lack of capability to collect selected cells. To enable the effective collection of highly metastatic cells invading across a matrix, we designed an open region connected directly to a space off the chip, resulting in convenient trypsinization and collection of the selected cells. Using this system, only one chip was required to yield enough selected cells for off-chip cell amplification, and *in vitro* and *in vivo* data showed that the cells undergoing off-chip amplification maintained the invasive phenotypes of the selected cells. Based on the effective collection, a subline with a high metastatic potential was obtained by only two selection cycles. We believe that this design facilitates cell collection not only for subline selection but also for downstream *in vitro* molecular change analysis and even *in vivo* behavior research. The majority of previously reported microfluidic systems have focused on invasive cell morphological observation and have not provided strong evidence of biological behavior or molecular changes. Our system offers a new avenue for the combination of microfluidics and other downstream techniques to investigate biological issues more deeply.

In summary, we developed a novel microfluidic system to successfully select a gastric cancer subline with high metastatic potential based on cell invasion differences by designing an open region for cell collection and a Petri dish-based liquid supply for long-term gradient maintenance. Apart from demonstrating highly effective selection, this system is simple, inexpensive and repeatable. Both *in vitro* and *in vivo* assays showed that the obtained subline cells had excessive metastatic ability. These cells could serve as a model for anticancer drug screening, metastasis mechanism research, and even new metastasis-related biomarker discovery via the investigation of molecular differences, such as differences in proteins and microRNA, between the parental and subline cells. In addition, this system might be further modified to perform real-time observation of the cell invasion process by combinational use with a live-cell imaging system.

## Material and Methods

### Cell culture and animals

Several cell lines were used in this study, including the human embryonic kidney cell line HEK-293, the human gastric cancer cell lines SGC-7901 and BGC-823, the human breast cancer cell line MDA-MB-231 and the rat glioma cell line C6. The cells were cultured in Dulbecco’s Modified Eagle’s Medium (DMEM, Thermo Fisher, Grand Island, NY, USA) supplemented with 10% fetal bovine serum (FBS, Sigma-Aldrich, St Louis, MO, USA) and 100 units/mL penicillin/streptomycin (Thermo Fisher) at 37 °C in a humidified atmosphere containing 5% carbon dioxide.

Five-week-old BALB/c nude mice (female) were purchased from Shanghai Slake Animal Ltd. (Shanghai, China) and had free access to food and water in an SPF-grade laboratory. The animal study protocols were approved and performed in accordance with the guidelines of the Ethical Committee of China Medical University.

### Design and fabrication of microfluidic system

The microfluidic system (Fig. 1B) consisted of a PDMS (Polydimethylsiloxane)-glass microfluidic device, a Petri dish (10 cm in diameter) and a syringe pump. According to tumorigenesis theory, tumor metastasis is a complex cascade of sequential steps, including cell invasion across the basal membrane, migration through the extracellular matrix, intravasation into the blood circulation and extravasation at distant organs. Among which, the initial and key step is to invade across the basal membrane. Accordingly, we designed a microfluidic device with various microchannels to mimic the invasion process and thus selected a highly metastatic subline based on different cell invasion abilities.

The PDMS-glass microfluidic device (Fig. 1A) contained four main microchannels, a medium channel, a cell culture channel, a matrix channel and an open region. Each channel was 200 μm in width and 45 μm in height. The medium channel was used for a continuous supply of culture medium through a single inlet connected to a syringe pump. The cell culture channel was used for cell seeding and culturing. The matrix channel was designed for Matrigel loading to mimic the *in vivo* cell invasion microenvironment. The adjacent channels were connected with each other by a series of narrow gaps (50 μm in length, 10 μm in width and 45 μm in height, the neighbored gaps were 90 μm apart). The length of channel was 1 cm. The open region, which was in direct connection with the space off the chip in the Petri dish, was fabricated for the collection of selected cells.

Because *in vivo* tumor metastasis is driven by chemotactic factor concentration gradients, here, we created a similar gradient using FBS as a chemotactic factor to mimic the *in vivo* environment. A low concentration of FBS (2%) was provided via perfusion of the medium into the medium channel using the syringe pump. A high concentration of FBS (10%) was supplied by placing the device into a Petri dish containing the medium. By the solute concentration-dependent diffusion across the gaps between channels, a vertical concentration gradient could be formed from the medium channel to the open region (Fig. 1B,C). To decrease the sharp shear force, multiple outlets were designed to connect with the medium channel.

A mask drawn using AutoCAD 10.0 was used to construct the polydimethylsiloxane (PDMS) microchannels by a soft lithography method. Negative photoresist SU-8 (MicroChem, Newton, MA, USA) was spun onto a silicon wafer. Sylgard 184 prepolymer (Dow Corning, Midland, MI, USA) was dispensed at a 10:1 ratio of polymer to curing agent onto the SU-8 mold. The PDMS was punched and cut vertically along the central axis of open region to create a cell collection region. Then, the PDMS was bonded with cover glass to form the microfluidic device.

### Establishment and maintenance of solute concentration gradient

We used FITC (fluorescein isothiocyanate, Sigma-Aldrich, USA) solution to investigate the concentration gradients. The Matrigel^TM^ (BD, USA) was used to mimic the base membrane *in vivo*. Matrigel diluted with PBS by 1:2 in volume was injected into the matrix channel and placed in 37 °C for gelation; this device was named the “Matrigel-loaded device”. The Matrigel-loaded device was then placed into the Petri dish containing 12 mL of FITC solution (0.1 mg/mL). PBS without FITC was pumped through the medium inlet at a speed of 3 μL/h to establish a vertical FITC concentration gradient from the medium channel to the open region. The concentration gradient was imaged by fluorescent microscopy over a series of time points. The fluorescent intensities of various channels at different time points were measured using ImageJ software.

### Cell viability assay

HEK-293 cells (5 × 10^6^ per mL) were injected into the cell culture channel of the Matrigel-loaded device. The device was placed in the Petri Dish containing 12 mL of DMEM supplemented with a high concentration of FBS (10%). DMEM with a low concentration FBS (2%) was pumped (3 μL/h) through the inlet continuously to establish the FBS concentration gradient and to prevent the channels from drying out. Then, the Petri dish with the device was placed in a cell incubator. After 3 and 5 days, the cells were stained with PI and calcein-AM (Thermo Fisher) according to the manufacturer’s instructions. Cell growth status was imaged by fluorescent microscopy.

### Evaluation of cell invasiveness

Cell suspensions (5 × 10^6^ per mL) of different cell lines (HEK-293, SGC-7901, BGC-823, MDA-MB-231 and C6) were seeded and cultured under the conditions described above. After having been driven by the concentration gradient for 3 days, the migration distance of the leading cells (i.e., the outermost cell of each gap that had migrated from the culture channel to the matrix channel or the open region) was measured to evaluate cell invasive potentials quantitatively using ImageJ. The mean value of eight gaps within one field was calculated. Five fields were counted for each group. Different number (3 × 10^6^, 6 × 10^6^ and 9 × 10^6^) of SGC-7901 cells was seeded and performed the same procedure described above to evaluate the invasiveness based on the invasion distance.

### MTS assay

Same number (2000 cells per well) of BGC-823 and MDA-MB-231 cells was seeded into the 96-well plates and cultured continuously for one to four days. 20 μL of MTS solution (Promega, USA) was added to each well in each time point. After 1 h of additional incubation, the absorbance at a wavelength of 490 nm was recorded by ELISA plate reader. Every five wells of one cell type were assessed in each time point. The mean values were used for cell proliferation measurement.

### Screening of sublines

Human gastric cancer SGC-7901 cells (named parental cells) were seeded into the culture channel (5 × 10^6^ per mL for each selection cycle) and driven to migrate along the gradient formed as described above (from 2% to 10% FBS). Four days later, cells that invaded to the open region were trypsinized. After breaking off the liquid supply, the device was washed and placed horizontally. 0.02% EDTA-0.25% trypsin solution was added on the glass substrate outside the open region and act upon the invasive cells by diffusion. When the cells in the open region became round, the device was slantwise placed into a Petri dish (6 cm in diameter) at the angle of 45° along its long axis, allowing an effective cell collection by rinsing downward, which could avoid the detachment of cells within other channels. Afterward, the cells were amplified for about ten days and denoted as SGC-7901/B1. The same density of SGC-7901/B1 cells were re-seeded into the cell culture channel and subjected to the same selection. The achieved subline cells were named SGC-7901/B2 and stored in liquid nitrogen for further characterization.

### Colony formation assay

Parental SGC-7901, SGC-7901/B1 and subline SGC-7901/B2 cells were suspended in 0.3% agar (Amresco, America), respectively. Cells (1000 cells/well) were seeded in a six-well plate containing an underlayer of 0.6% agar and incubated at 37 °C in a humidified atmosphere of 5% CO_2_. After two weeks of incubation, colonies containing more than 50 cells were counted. The colony formation efficiency was measured as the average percentage of three wells.

### Transwell invasion assay

Diluted Matrigel (100 μL) was loaded on the membranes of the transwell chambers (Corning, America). After gelation, the chambers were placed above the lower well containing DMEM supplemented with 10% FBS. Parental SGC-7901, SGC-7901/B1 and subline SGC-7901/B2 cells with different passages suspended in serum-free DMEM were seeded onto the upper chambers, respectively. After 24 h, the invading cells present on the lower surface of each chamber membrane were fixed and stained with Trypan Blue. Six fields were examined for each well, and cells were counted for evaluation.

### Tumorigenicity assays

Tumorigenicity assays were performed to evaluate the *in vivo* proliferation and metastatic potential of the subline cells. Parental or subline cell suspensions (2 × 10^6^) were injected in the armpits of BALB/c nude mice (nine mice per group) subcutaneously. When the largest palpable subcutaneous tumor reached 1.5 cm in diameter, the mice were euthanized. The subcutaneous tumors and a series of metastasis-involved organs (including lungs, liver, kidneys and lymph nodes) were excised. The subcutaneous tumors were weighed. The metastatic foci in different organs were evaluated on a gross level and by microscopy. Organs containing metastatic foci were sectioned and stained with hematoxylin and eosin. Nude mice (seven mice per group) were injected with 1 × 10^6^ parental or subline cells through the tail vein and were observed continuously for survival analysis. The period of survival was recorded and analyzed statistically.

### Western blot analysis

Parental and subline cells were subjected to gene expression profiling analysis by microarrays (Agilent human 4 × 44 K gene expression microarrays, USA). To confirm the analysis of transcriptional profiling, we chose two metastasis-related proteins, E-cadherin and Smad3, for further protein expression analysis. Extracts of protein from the parental and the subline cells were prepared according to standard procedures. Protein samples were subjected to SDS-polyacrylamide gel electrophoresis and transferred to a polyvinylidene fluoride (PVDF) membrane. After blocking, the membrane was incubated with the primary antibodies (Cell Signaling, USA) overnight at 4 °C. After repeated washing, the membrane was incubated with the secondary horseradish peroxidase-conjugated antibody (1:5000) for 2 h at room temperature. Immunoreactive bands were detected using the ECL method.

### Statistical analysis

The statistical data obtained are presented as the mean value and standard deviation. Student *t-test* analysis was used in SPSS 13.0 to determine significant differences between groups, and *P* values less than 0.05 were considered statistically significant. Survival rate estimation and median survival durations were determined using the Kaplan-Meier method.

## Additional Information

**How to cite this article**: Chen, Z.-z. *et al*. Establishment of a gastric cancer subline with high metastatic potential using a novel microfluidic system. *Sci. Rep.* **6**, 38376; doi: 10.1038/srep38376 (2016).

**Publisher's note:** Springer Nature remains neutral with regard to jurisdictional claims in published maps and institutional affiliations.

## Supplementary Material

Supplementary Information
