# Supplementary material for: Establishment of a gastric cancer subline with high metastatic potential using a novel microfluidic system
Source: Sci Rep. 2016 Dec 5;6:38376. doi: 10.1038/srep38376 (PMC5137147; doi:10.1038/srep38376)
Supplement: Supplementary Information [file srep38376-s1.doc]

**Establishment of a gastric cancer subline with high metastatic potential using a novel microfluidic system**

**Author names and affiliations**

Zhezhou Chen^1^, Wanming Li^1^, Yu Zhang^1^, Min Yu^1^, Lianfeng Shan^2^, Dezheng Yuan^1^, Furong Liu^1^, Jin Fang^1*^

^1^Department of Cell Biology, Key Laboratory of Cell Biology, Ministry of Public Health, and Key Laboratory of Medical Cell Biology, Ministry of Education, China Medical University, Shenyang 110122, PR China

^2^Department of Mathematics, China Medical University, Shenyang 110122, PR China

**Supplementary Data 1**

**Figure S1. Invasiveness analysis of SGC-7901 in different cell densities using the microfluidic system.** (A) Different number of SGC-7901 were seeded into the culture channel. 24 h later, the invasion distances of leading cells were visualized by the microscope. (B) The corresponding invasion distances were analyzed quantitatively (original magnification ×100). Each independent experiment was repeated three times.

**Supplementary Data 2**

**Figure S2. Proliferation abilities of two cell lines evaluated by MTS assay.** Same number of BGC-823 and MDA-MB-231 cells were seeded into the 96-well plates and cultured. The MTS solution was added at different time points, respectively. The mean values of absorbance were recorded and used for proliferation measurement. Each independent experiment was repeated three times.

**Supplementary Data 3**

**Figure S3. Analysis of invasive ability of SGC-7901/B2 from different passages by transwell.** Transwell invasion assay was performed for the parental SGC-7901 and subline SGC-7901/B2 cells with four and ten passages. The invading cells were stained by Trypan Blue (A) and counted for quantitative analysis (B). Each independent experiment was repeated three times.
